# Supplementary material for: SNVstory: inferring genetic ancestry from genome sequencing data
Source: BMC Bioinformatics. 2024 Feb 20;25:76. doi: 10.1186/s12859-024-05703-y (PMC10877842; doi:10.1186/s12859-024-05703-y)
Supplement: Supplementary file 1 — Additional file 1: Supplemental methods, figures, and tables. [file 12859_2024_5703_MOESM1_ESM.pdf]

-- Supplemental Materials and Methods --

SNVstory: Inferring Genetic Ancestry from Genome Sequencing Data

Audrey E. Bollas[1], Andrei Rajkovic[1], Defne Ceyhan[1], Jeffrey B. Gaither[1], Elaine R. Mardis[1,2], Peter White[1,2,*]

[1] The Steve and Cindy Rasmussen Institute for Genomic Medicine, The Abigail Wexner Research Institute, Nationwide Children's Hospital, Columbus, OH USA

[2] Department of Pediatrics, The Ohio State University College of Medicine, Columbus, OH USA

* Corresponding Author:

Peter White, Ph.D.

The Institute for Genomic Medicine

The Abigail Wexner Research Institute

Nationwide Children's Hospital

575 Children's Crossroad

Columbus, OH 43215

Phone: +1 (614) 355-2671

Email: peter.white@nationwidechildrens.org

SGDP quality control

Aligned reads and VCFs for each sample were processed with our in-house quality control pipeline. Summarized results are available in **Table S1**, which includes genome sequencing coverage, number of mapped and unmapped reads, number of variants, and contamination status.

One sample (LP6005441-A09) was flagged for possible contamination. There were no other indications of poor quality based on the above metrics. Notably, ~98% of the reads were aligned to the human reference genome, reducing the likelihood the flag is due to bacterial contamination.

Removal of related samples

SGDP: KING provided a kinship coefficient for each pairwise sample. Using the coefficient cutoff >= 0.0442 for relatedness, 78 pairwise calculations were returned. However, 74 of these stemmed from one sample (LP6005441-A09) with a high coefficient to the 74 other samples. This is the same sample that was flagged for contamination in quality control. Two pairwise calculations resulted from one sample (LP6005441-G06) related to two others. The last two calculations were singleton relatedness, so the sample in the first ID column was chosen to be removed. Four samples (LP6005441-A09, LP6005441-G06, LP6005441-B12, LP6005443-E11) were removed from further analysis based on relatedness. Additionally, one more sample (LP6005443-C01) was removed at this step because it was not present in the metadata and therefore did not have an ancestry label.

Benchmarking

ADMIXTURE: Plink 2 was used to create input .bed files with the command: *"plink2 –vcf <input_vcf> --make-bed –output <output_prefix>"*. We also created .pops file which specify the ancestries of the reference samples and designates which samples should be estimated with a "-". We ran ADMIXTURE with the supervised learning mode enabled by using the flag "*—supervised*", and with default parameters. To determine the Q estimate population labels, we aggregated each reference population's Q estimates by the mean and returned the label maximum mean Q value. The Q values for the remaining samples of unknown ancestry were used for further analysis.

RFMIX: Beagle 5.4 was used to phase the input VCFs. GRCh38 genetic maps were obtains from https://bochet.gcc.biostat.washington.edu/beagle/genetic_maps/. Imputation was not performed during phasing. RFMIX was run using default parameters. RFMIX returns Q value results for each chromosome separately. To obtain global ancestry assignment for the entire sample, we aggregated the Q values across all chromosomes by the mean, to return one Q value for each sample for each population.

For validation, we use both the 1kGP and SGDP datasets as input. Because both methods require the user to input reference data, we used one dataset for reference and one dataset for testing. For example, *"rfmix -f <1kGP_test.bcf> -r <SGDP_ref.bcf> -m <SGDP_sample_map.txt> -g <genetic_map.txt> -o <1kGP.chr1> --chromosome=chr1"*. To provide a fair comparison, we use the same samples for testing across all three methods.

Supplemental Figures

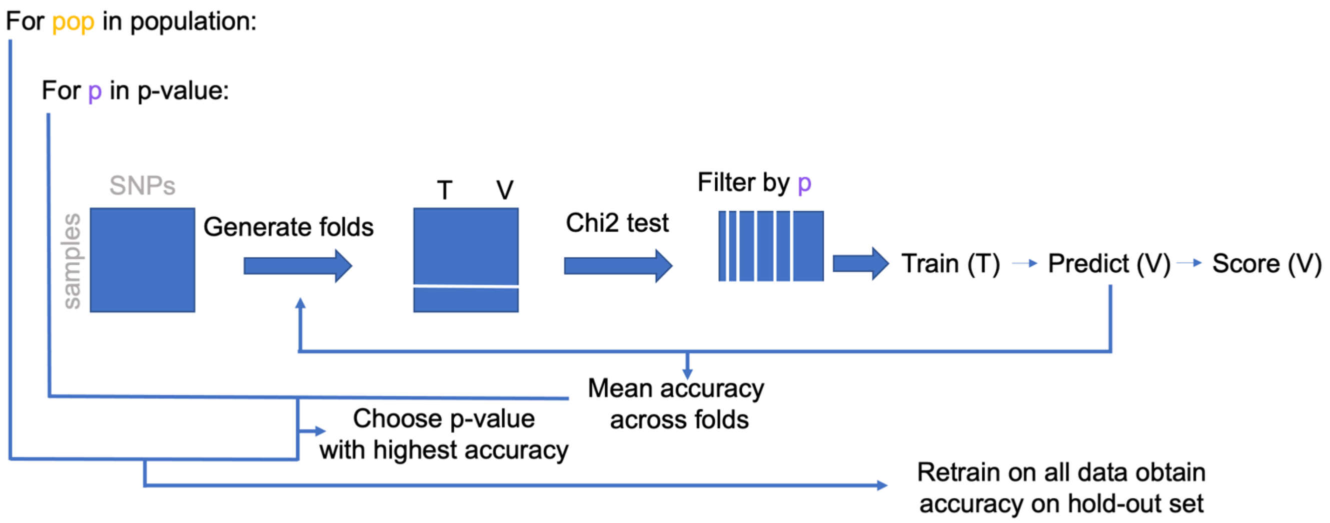

Figure S1. gnomAD model cross-validation design. Cross-validation design for gnomAD model to select SNP p-value threshold and population size.

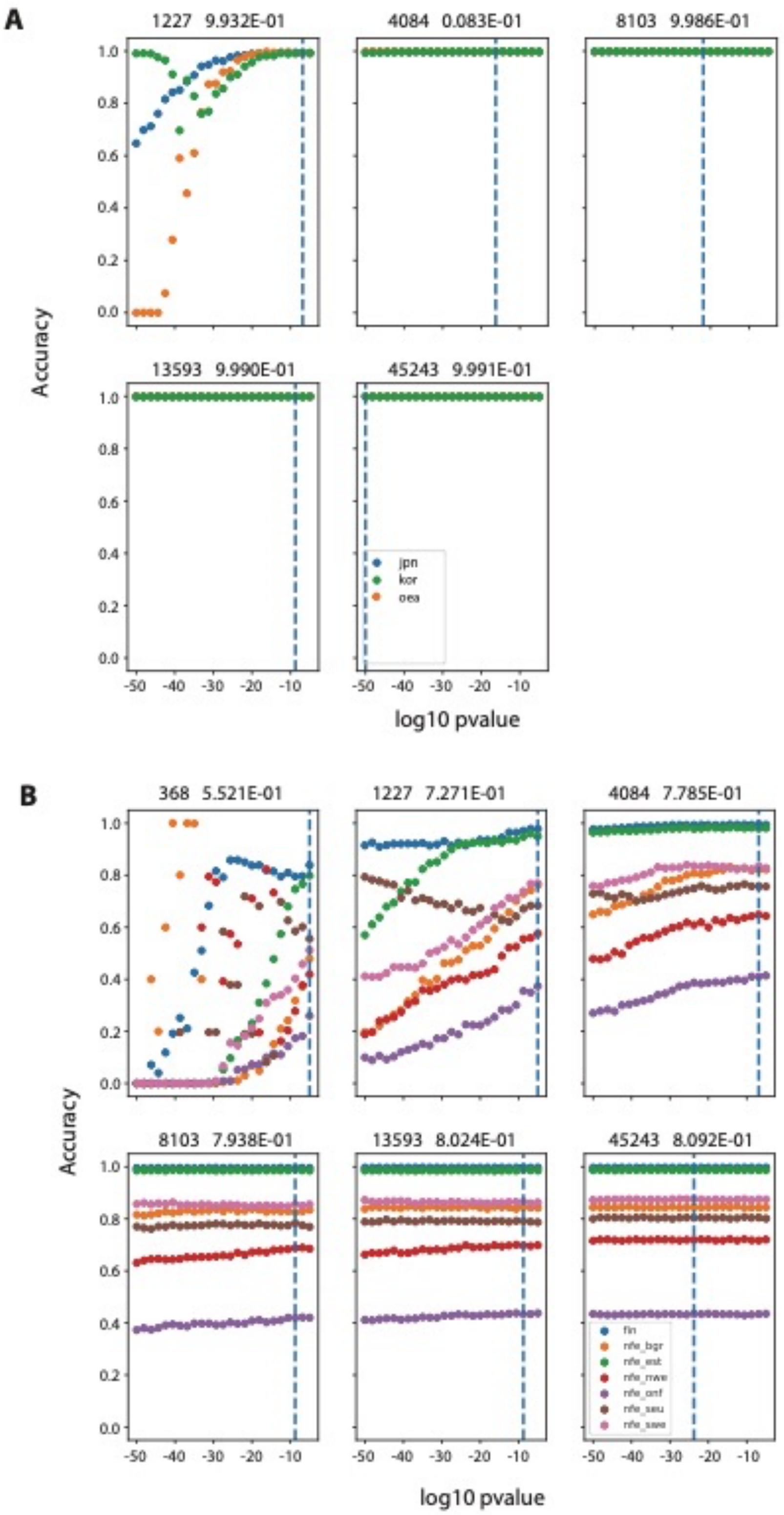

Figure S2. P-value thresholds. P-value thresholds for each population size in the East Asia (**A**) and Europe (**B**) sub-continental models. Each population is represented in a separate plot, with the leading number as the number of individuals. Each data point represents a cross-validation for a specific p-value. The blue dashed line indicates the p-value that yields the maximum accuracy.

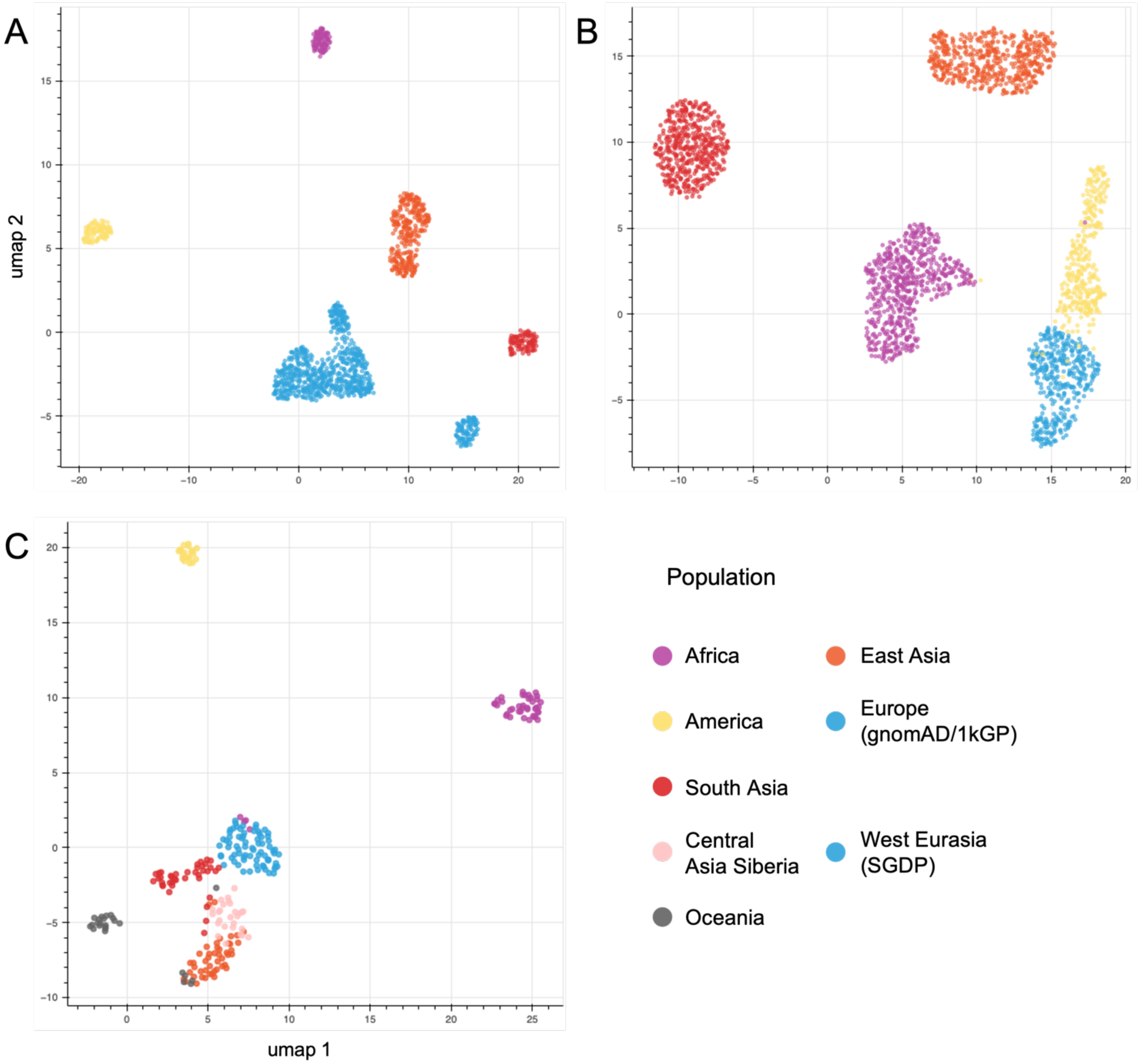

Figure S3. Uniform Manifold Approximation and Projection (UMAP) dimensionality reduction of population data. is a dimensionality reduction Visualization of UMAP of gnomAD (**A**), 1kGP (**B**), and SGDP (**C**).

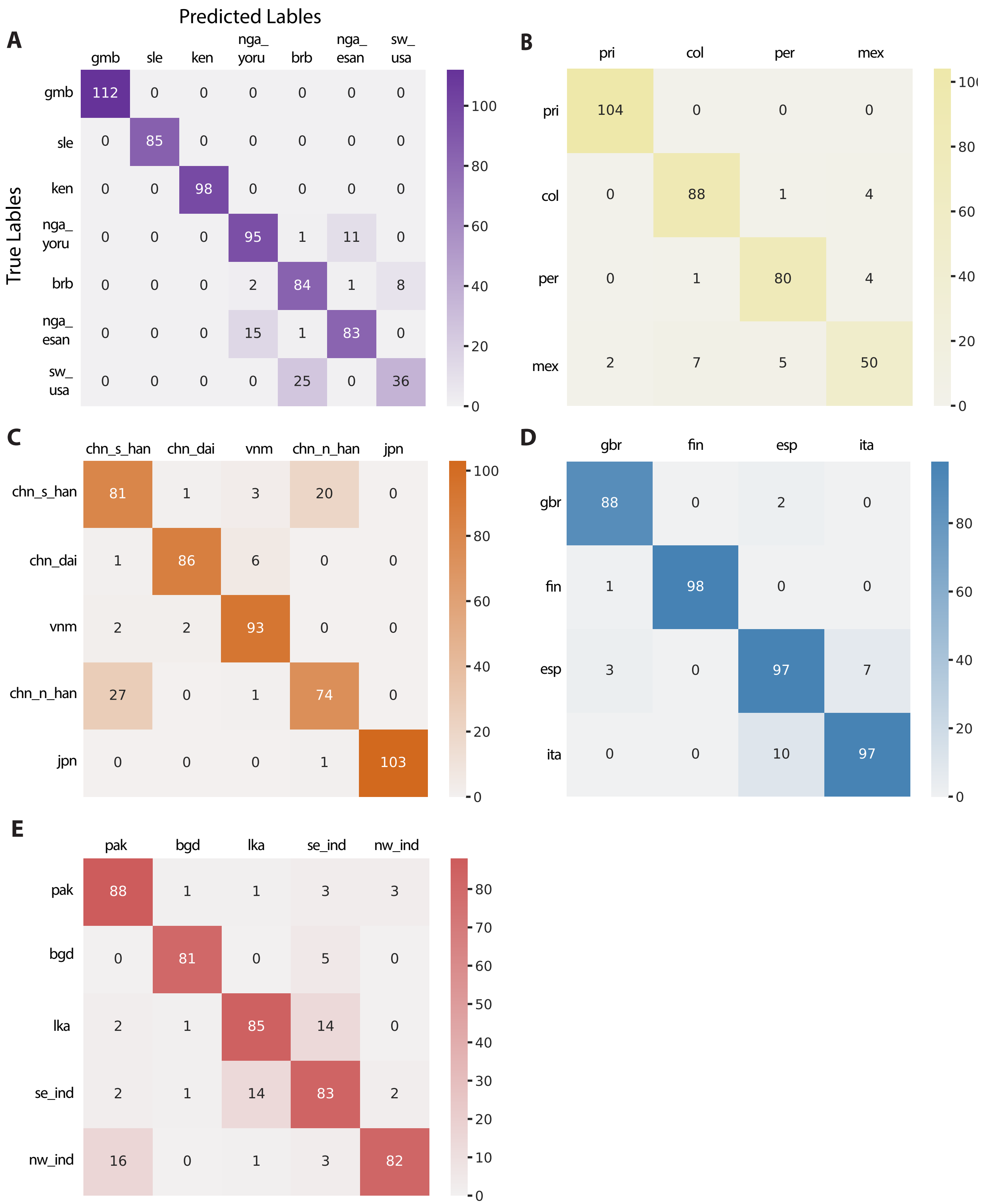

Figure S4. 1000 Genomes Project data cross-validation. 1kGP sub-continental cross-validation results for Africa **(A)**, America **(B)**, East Asia **(C)**, Europe **(D)**, and South Asia **(E)**.

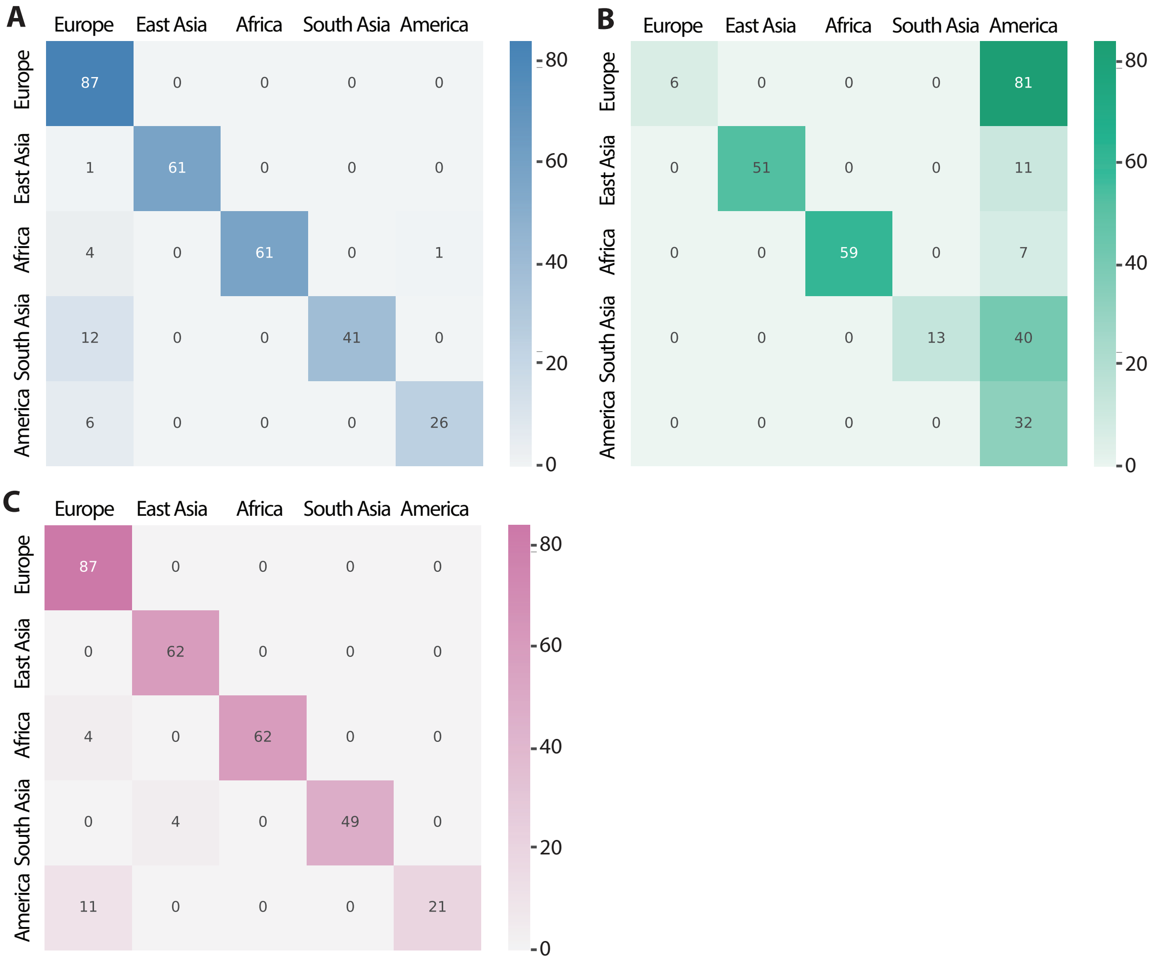

Figure S5. Continental model Performance Comparison. Validation data results using SNVstory **(A)**, ADMIXTURE **(B)**, and RFMIX **(C)**.

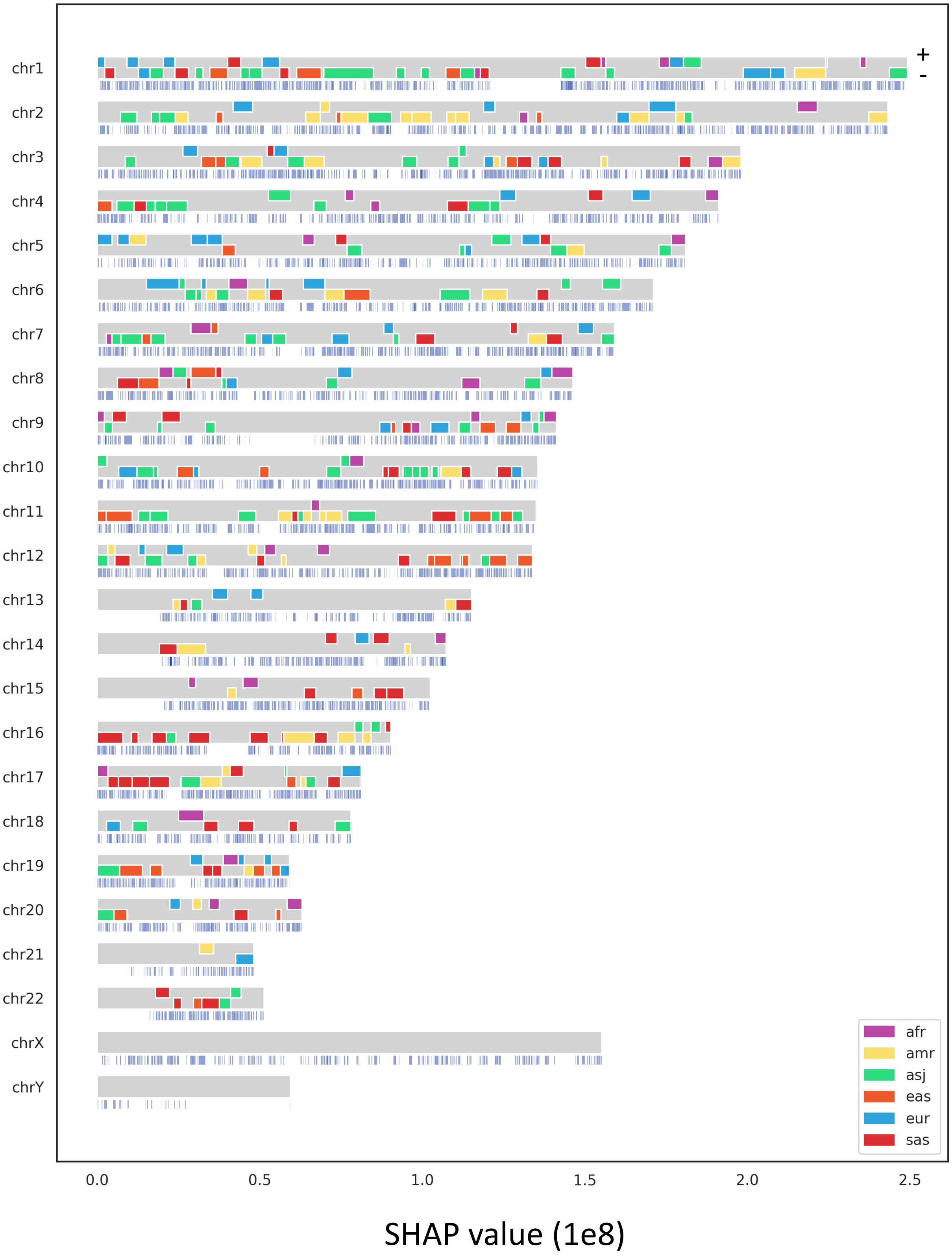

Figure S6. Ancestry inference is impacted by specific cytogenetic locations. SHAP values for an African individual, aggregated by cytolocation. Each chromosome is split horizontally for positive/negative (upper/lower half) SHAP values.

Supplemental Tables

Table S1. Quality control of the Simons Genome Diversity Project (SGDP).

SDGP Sample	Contam Status	WGS Coverage	Mapped Reads	Unmapped Reads	Total Variants
LP6005442-G09	CLEAN	40.7	1,262,249,988	11,762,044	4,800,544
LP6005443-G08	CLEAN	42.5	1,330,248,287	20,665,942	6,042,069
LP6005441-H09	CLEAN	37.2	1,158,952,930	16,261,254	4,752,562
LP6005443-H07	CLEAN	43.0	1,339,534,642	22,330,734	4,560,297
LP6005519-H03	CLEAN	33.4	1,035,577,472	13,662,192	4,725,473
SS6004475	CLEAN	37.7	1,177,556,638	16,623,166	5,583,702
LP6005519-E06	CLEAN	36.5	1,140,356,265	325,628,526	4,617,868
LP6005677-E01	CLEAN	41.4	1,295,331,962	19,492,150	4,454,912
LP6005592-G03	CLEAN	42.8	1,347,919,725	36,281,340	4,668,161
LP6005519-G04	CLEAN	43.6	1,344,463,524	18,284,228	4,745,692
LP6005677-C03	CLEAN	41.6	1,285,853,306	18,412,486	4,689,460
LP6005442-G10	CLEAN	37.3	1,156,756,287	14,613,318	5,593,768
LP6005441-G11	CLEAN	36.9	1,143,079,349	5,991,438	4,685,322
LP6005443-E11	CLEAN	43.1	1,348,966,959	18,151,350	4,522,505
LP6005442-E12	CLEAN	39.1	1,204,425,458	12,923,730	4,727,303
LP6005441-H10	CLEAN	36.3	1,124,288,538	20,920,636	4,701,613
LP6005441-F12	CLEAN	36.3	1,134,555,930	36,620,648	4,564,465
LP6005443-F10	CLEAN	42.5	1,329,824,008	20,461,184	4,710,973
LP6005442-F11	CLEAN	36.1	1,119,767,819	4,918,592	5,641,725
LP6005441-D05	CLEAN	64.0	1,985,830,483	23,364,930	4,756,501
LP6005443-D03	CLEAN	42.6	1,311,405,446	16,603,604	4,517,445
LP6005441-F03	CLEAN	38.2	1,186,740,411	9,838,690	4,784,910
LP6005443-F01	CLEAN	44.3	1,373,775,270	30,560,384	4,640,332
LP6005442-F02	CLEAN	35.6	1,125,094,395	30,123,580	4,649,458
LP6005441-B07	CLEAN	35.7	1,106,956,798	23,100,678	4,666,271
LP6005443-E02	CLEAN	41.2	1,278,471,645	15,048,798	5,645,378
LP6007068-A01	CLEAN	39.7	1,230,928,969	15,974,932	4,596,190
LP6005441-A08	CLEAN	35.7	1,111,781,198	6,995,536	5,840,761
LP6005443-A06	CLEAN	41.9	1,302,422,843	23,310,678	4,670,000
LP6005441-G02	CLEAN	40.5	1,260,447,750	3,480,554	5,927,550
LP6005441-C06	CLEAN	77.0	2,400,408,252	33,226,002	4,739,486
LP6005442-G01	CLEAN	38.5	1,205,179,793	47,779,200	4,621,437
LP6005443-C04	CLEAN	37.3	1,153,686,523	15,757,482	4,632,982
LP6005441-C11	CLEAN	33.7	1,043,303,633	14,872,158	4,584,412
LP6005442-C10	CLEAN	32.5	1,006,267,088	5,215,394	4,631,662
LP6005442-A12	CLEAN	35.9	1,107,040,021	3,061,216	4,737,618
LP6005441-D10	CLEAN	39.1	1,209,503,202	5,117,812	4,804,262
LP6005442-B11	CLEAN	39.9	1,234,506,521	7,070,738	4,699,981
LP6005443-B10	CLEAN	42.4	1,314,270,053	21,545,940	4,701,644
LP6005441-B12	CLEAN	33.0	1,021,186,165	9,878,272	4,278,987
LP6005441-D01	CLEAN	39.4	1,226,689,973	8,235,916	4,605,831
LP6005442-B02	CLEAN	33.4	1,035,119,894	8,411,216	5,644,168
LP6005443-B01	CLEAN	36.8	1,142,307,257	22,109,852	4,634,270
LP6005441-B03	CLEAN	38.7	1,201,802,234	9,756,630	4,642,729
SS6004479	CLEAN	36.7	1,147,560,269	18,316,680	4,432,381
LP6005442-A03	CLEAN	39.1	1,227,362,695	6,449,118	4,684,239
LP6005441-A04	CLEAN	37.8	1,176,408,024	9,199,466	4,376,063
LP6005443-A02	CLEAN	45.9	1,424,442,510	16,739,198	4,676,823
LP6005441-C02	CLEAN	38.7	1,200,678,458	18,557,106	4,639,937
LP6005519-D03	CLEAN	43.1	1,333,356,610	42,739,202	4,706,967
LP6005592-B04	CLEAN	31.9	989,951,011	15,751,650	4,704,697
LP6005519-B05	CLEAN	45.3	1,396,376,920	17,267,680	4,689,197
SS6004471	CLEAN	36.0	1,125,698,922	16,171,228	5,808,618
LP6005592-E01	CLEAN	42.9	1,336,964,387	48,961,714	4,662,546
LP6005519-A06	CLEAN	45.9	1,419,414,673	155,629,922	4,758,847
LP6005519-C04	CLEAN	51.1	1,574,571,003	16,750,216	4,745,038
LP6005592-C03	CLEAN	38.2	1,190,643,628	22,617,840	5,883,394
LP6005441-D09	CLEAN	37.0	1,144,988,946	3,264,478	4,644,766
LP6005443-D07	CLEAN	47.3	1,460,444,224	23,709,464	4,605,046
LP6005442-H04	CLEAN	37.4	1,154,862,260	5,481,620	4,679,693
LP6005442-D08	CLEAN	35.6	1,102,634,219	6,691,444	4,605,710
LP6005441-H05	CLEAN	37.5	1,165,766,113	10,910,632	4,678,354
LP6005443-H03	CLEAN	46.3	1,424,834,713	14,075,182	4,588,853
LP6005441-F07	CLEAN	36.2	1,119,454,463	5,342,016	5,644,017
LP6005443-B09	CLEAN	33.5	1,048,507,395	285,867,232	5,465,130
SS6004480	CLEAN	34.2	1,095,574,277	221,627,670	5,424,671
LP6005443-E06	CLEAN	35.9	1,114,935,800	19,872,072	5,434,444
LP6005441-E08	CLEAN	36.1	1,119,417,095	5,468,570	4,607,014
LP6005442-E07	CLEAN	34.3	1,062,487,438	4,396,880	4,471,932
LP6005443-G04	CLEAN	43.2	1,330,809,386	19,560,904	4,706,877
LP6005441-G06	CLEAN	41.3	1,288,148,142	37,146,160	4,328,488
LP6005443-C08	CLEAN	40.2	1,255,501,499	23,341,924	4,574,984
LP6005442-A10	CLEAN	33.0	1,020,048,219	4,898,890	5,575,903
LP6005441-A11	CLEAN	34.2	1,067,390,948	27,129,442	5,904,921
SS6004468	CLEAN	40.2	1,244,318,006	20,394,498	4,603,840
LP6005441-B10	CLEAN	41.7	1,301,577,629	51,310,612	4,580,291
LP6005441-B01	CLEAN	32.7	1,014,946,834	6,519,280	4,648,161
SS6004477	CLEAN	40.0	1,239,112,059	14,157,848	4,667,922
LP6005677-G01	CLEAN	35.4	1,096,133,813	20,873,480	5,597,054
LP6005442-G12	CLEAN	38.2	1,178,076,719	2,168,208	4,669,905
LP6005519-D01	CLEAN	53.8	1,662,030,852	19,964,182	4,508,354
LP6005619-C01	CLEAN	46.8	1,448,665,452	18,352,140	4,941,014
LP6005443-G11	CLEAN	40.8	1,274,128,818	18,528,170	4,570,052
LP6005592-B02	CLEAN	34.1	1,067,115,881	98,666,988	4,735,596
LP6005442-H11	CLEAN	34.8	1,077,057,211	9,597,448	5,713,087
LP6005519-A04	CLEAN	47.4	1,464,341,163	25,165,606	4,797,234
LP6005441-H12	CLEAN	36.8	1,145,442,618	28,756,920	4,630,464
LP6005592-C01	CLEAN	46.5	1,438,681,470	11,308,430	4,667,578
LP6005441-D07	CLEAN	38.2	1,180,304,681	7,412,354	4,667,053
LP6005443-D05	CLEAN	44.3	1,368,781,808	22,006,492	4,674,867
LP6005441-H03	CLEAN	36.2	1,120,922,320	7,094,878	4,736,210
LP6005443-H01	CLEAN	46.7	1,445,065,076	14,811,736	4,699,387
LP6005442-B08	CLEAN	37.3	1,151,424,280	4,303,654	4,704,897
LP6005441-F05	CLEAN	59.0	1,831,678,118	19,177,334	4,793,389
LP6005443-F03	CLEAN	45.9	1,414,682,396	13,477,462	4,560,536
LP6005442-F04	CLEAN	34.0	1,048,408,870	3,541,976	4,662,492
LP6005443-B07	CLEAN	42.3	1,316,970,946	19,245,530	4,637,312
LP6005441-B09	CLEAN	39.2	1,223,108,548	19,119,584	4,693,542
LP6005441-E06	CLEAN	34.3	1,067,369,815	4,114,342	4,586,482
LP6005443-A08	CLEAN	41.6	1,291,015,593	22,921,610	4,585,715
LP6005443-G02	CLEAN	40.0	1,242,979,757	14,342,566	5,831,031
LP6005442-C07	CLEAN	33.9	1,051,479,126	4,594,242	4,542,984
LP6005441-G04	CLEAN	40.8	1,274,534,690	5,987,382	4,642,544
LP6005441-C08	CLEAN	32.2	999,666,456	6,431,838	4,563,601
LP6005442-G03	CLEAN	32.6	1,006,593,151	7,508,012	4,593,000
LP6005443-C06	CLEAN	49.7	1,535,554,564	16,307,634	4,708,809
LP6005442-E10	CLEAN	38.4	1,186,911,315	18,991,432	4,616,992
LP6005441-D12	CLEAN	35.7	1,109,136,483	15,153,452	4,659,939

SDGP Sample	Contam Status	WGS Coverage	Mapped Reads	Unmapped Reads	Total Variants
LP6005442-D11	CLEAN	35.7	1,103,307,273	4,008,178	4,699,139
LP6005441-F10	CLEAN	35.0	1,086,013,686	6,597,820	4,366,592
LP6005441-D03	CLEAN	35.1	1,092,220,299	6,749,402	4,747,202
LP6005443-D01	CLEAN	46.5	1,445,556,525	23,181,700	4,774,919
LP6005442-D02	CLEAN	35.8	1,115,800,992	15,050,886	4,704,531
LP6005442-B04	CLEAN	39.7	1,226,000,384	8,406,078	4,711,134
LP6005441-F01	CLEAN	37.9	1,180,064,626	13,343,790	5,682,979
LP6005443-B03	CLEAN	40.4	1,247,046,405	14,583,882	4,593,272
LP6005441-B05	CLEAN	37.3	1,167,725,600	4,404,978	4,646,028
LP6005441-E02	CLEAN	40.1	1,253,548,915	6,703,738	4,675,873
LP6005441-A06	CLEAN	63.3	1,977,971,732	37,045,532	4,711,513
LP6005443-C02	CLEAN	47.8	1,479,338,009	19,293,686	4,720,408
LP6005519-D05	CLEAN	48.8	1,507,251,417	16,651,710	4,764,309
LP6005592-D04	CLEAN	38.2	1,187,872,233	28,486,912	4,565,616
LP6005519-F03	CLEAN	42.2	1,301,033,117	27,159,504	4,715,940
LP6005677-B02	CLEAN	45.1	1,392,992,336	15,098,772	4,725,591
SS6004473	CLEAN	37.0	1,155,649,637	15,603,506	5,977,758
LP6005519-E04	CLEAN	45.3	1,396,350,189	17,395,674	4,788,857
LP6005677-A03	CLEAN	41.2	1,272,446,698	16,015,722	4,703,787
LP6005592-G01	CLEAN	51.4	1,603,618,597	17,541,546	4,772,351
LP6005519-G02	CLEAN	55.9	1,721,488,225	24,427,458	4,535,784
LP6005519-C06	CLEAN	35.8	1,102,432,594	63,456,884	4,594,162
LP6005592-C05	CLEAN	47.6	1,478,964,526	58,188,648	6,103,191
LP6005443-D09	CLEAN	36.6	1,138,239,384	130,977,270	4,450,868
LP6005441-H07	CLEAN	41.9	1,295,466,348	10,854,856	4,485,105
LP6005443-H05	CLEAN	45.8	1,426,680,573	18,194,140	4,648,348
LP6005441-F09	CLEAN	37.7	1,163,927,943	3,238,024	4,621,562
LP6005443-F07	CLEAN	45.2	1,398,903,760	24,457,368	4,585,199
LP6005443-E08	CLEAN	32.4	1,009,137,440	23,606,308	4,562,567
LP6005441-G08	CLEAN	35.0	1,083,092,028	3,855,846	4,858,228
LP6005442-G07	CLEAN	34.9	1,082,063,803	4,973,612	4,643,731
LP6005441-A10	CLEAN	31.0	966,347,149	33,307,806	4,447,623
SS6004467	CLEAN	36.1	1,128,475,956	14,428,270	4,577,047
LP6005442-H09	CLEAN	31.6	977,133,554	3,704,706	4,745,820
LP6005443-H08	CLEAN	36.9	1,151,950,457	196,990,262	5,463,889
LP6005677-D03	CLEAN	43.6	1,359,738,381	35,252,370	5,944,348
LP6005519-H04	CLEAN	45.4	1,401,822,414	18,705,000	4,754,640
LP6005592-H03	CLEAN	42.1	1,316,986,149	24,378,182	4,632,500
LP6005519-F06	CLEAN	47.1	1,450,150,598	71,339,004	4,648,256
LP6005677-F01	CLEAN	39.1	1,208,193,466	18,396,040	4,536,684
SS6004476	CLEAN	34.1	1,066,078,037	16,319,024	4,345,067
LP6005441-A01	CLEAN	38.2	1,191,433,961	3,938,862	4,663,531
LP6005442-G11	CLEAN	37.3	1,156,554,145	12,048,166	5,652,601
LP6005441-G12	CLEAN	38.1	1,195,868,703	40,288,410	4,592,335
LP6005592-B01	CLEAN	46.0	1,425,188,330	12,762,458	4,689,728
LP6005442-H10	CLEAN	36.1	1,119,742,294	3,289,290	5,612,639
LP6005441-H11	CLEAN	36.2	1,116,883,646	8,726,300	4,730,904
LP6005592-A02	CLEAN	42.1	1,311,189,612	58,990,358	4,646,622
LP6005443-F11	CLEAN	43.2	1,341,446,350	18,215,854	4,513,039
LP6005619-B01	CLEAN	43.4	1,346,314,879	19,067,896	4,892,545
LP6005442-F12	CLEAN	36.5	1,126,712,182	4,213,256	4,690,962
LP6005441-D06	CLEAN	38.7	1,206,778,809	27,912,606	4,683,929
LP6005443-D04	CLEAN	44.5	1,372,867,029	18,606,870	4,605,102
LP6005442-H01	CLEAN	37.4	1,163,591,710	45,390,816	4,651,379
LP6005441-H02	CLEAN	40.2	1,249,334,718	3,144,430	5,918,829
LP6005441-F04	CLEAN	42.7	1,323,391,958	15,355,078	4,699,153
LP6005443-F02	CLEAN	35.6	1,104,531,313	22,170,774	5,734,873
LP6005443-B06	CLEAN	45.7	1,415,923,124	20,453,864	4,627,480
LP6005441-B08	CLEAN	35.3	1,099,892,814	7,878,484	5,862,990
LP6005442-A08	CLEAN	37.5	1,164,284,388	4,159,752	4,673,973
LP6005441-E05	CLEAN	61.1	1,896,386,857	28,031,824	4,772,893

SDGP Sample	Contam Status	WGS Coverage	Mapped Reads	Unmapped Reads	Total Variants
LP6005441-A09	POSSIBLE	38.1	1,187,227,875	26,420,932	4,886,792
LP6007069-A01	CLEAN	39.3	1,221,587,453	17,939,764	4,632,495
LP6005442-E04	CLEAN	37.4	1,152,835,573	9,270,398	4,749,026
LP6005443-A07	CLEAN	46.9	1,457,836,373	18,944,650	4,731,224
LP6005443-G01	CLEAN	45.3	1,403,220,958	19,306,860	4,643,707
LP6005441-G03	CLEAN	39.7	1,233,381,698	6,314,912	4,716,575
LP6005441-C07	CLEAN	39.5	1,225,959,743	5,967,888	4,767,034
LP6005442-G02	CLEAN	36.9	1,141,165,150	21,016,368	4,669,632
LP6005442-C11	CLEAN	34.4	1,064,477,369	3,763,914	4,652,551
LP6005441-E10	CLEAN	39.8	1,230,537,681	9,169,950	4,510,575
LP6005443-A12	CLEAN	41.9	1,309,088,067	15,477,282	4,536,851
LP6005441-D11	CLEAN	32.2	998,395,493	5,227,130	4,672,253
LP6005442-D10	CLEAN	36.1	1,116,528,108	5,281,504	4,647,658
LP6005442-B12	CLEAN	31.4	967,412,506	7,037,338	4,726,003
LP6005441-D02	CLEAN	35.7	1,106,171,759	11,118,414	4,631,967
LP6005442-D01	CLEAN	33.8	1,052,950,376	29,319,140	4,603,736
LP6005442-B03	CLEAN	34.7	1,074,891,747	6,719,712	4,646,334
LP6005443-B02	CLEAN	46.6	1,443,782,013	16,288,152	4,774,103
LP6005441-B04	CLEAN	38.0	1,180,899,055	14,815,428	4,451,798
LP6005442-A04	CLEAN	31.4	971,659,106	4,958,756	4,628,726
LP6005441-A05	CLEAN	40.6	1,272,721,646	5,239,542	4,611,507
LP6005443-A03	CLEAN	43.1	1,329,053,391	13,244,636	4,709,104
LP6005442-C02	CLEAN	34.8	1,079,534,133	8,558,518	4,653,453
LP6005441-C03	CLEAN	32.5	1,010,813,709	7,826,818	4,631,477
LP6005443-C01	CLEAN	43.7	1,331,747,311	159,690	4,194,954
LP6005519-D04	CLEAN	46.4	1,429,086,825	16,841,296	4,768,210
LP6005592-D03	CLEAN	46.0	1,424,980,004	22,238,932	4,714,897
LP6005592-F01	CLEAN	38.7	1,204,731,058	29,852,212	4,669,967
LP6005519-B06	CLEAN	52.8	1,637,226,609	70,773,990	4,738,362
LP6005677-B01	CLEAN	33.6	1,041,214,917	32,802,938	4,650,580
SS6004472	CLEAN	40.8	1,271,107,104	19,137,898	4,530,348
LP6005592-E02	CLEAN	44.1	1,375,788,899	57,020,466	4,621,445
LP6005677-A02	CLEAN	36.1	1,113,673,103	19,676,978	4,685,163
LP6005519-C05	CLEAN	46.1	1,422,302,506	17,525,656	4,763,309
LP6005443-D08	CLEAN	40.0	1,241,357,944	23,185,730	4,591,268
LP6005442-D09	CLEAN	39.1	1,206,242,553	3,331,738	5,226,522
LP6005441-H06	CLEAN	36.2	1,131,270,507	7,571,940	4,355,786
LP6005441-F08	CLEAN	41.8	1,291,274,226	2,759,948	4,709,515
LP6005443-F06	CLEAN	43.8	1,364,131,541	22,610,962	5,471,527
LP6005443-E07	CLEAN	44.8	1,393,852,473	29,423,186	4,527,657
LP6005441-E09	CLEAN	35.6	1,112,691,088	7,246,164	4,608,462
LP6005443-G05	CLEAN	42.9	1,328,871,902	19,406,712	4,597,908
LP6005441-G07	CLEAN	34.8	1,084,053,113	7,505,332	4,513,208
LP6005443-C09	CLEAN	34.1	1,065,770,844	241,055,184	4,730,901
LP6005441-C10	CLEAN	37.1	1,150,769,664	22,992,122	4,709,018
LP6005442-A11	CLEAN	33.0	1,022,569,614	1,721,568	4,616,347
LP6005441-A12	CLEAN	38.6	1,190,637,534	5,519,996	4,338,821
SS6004469	CLEAN	34.5	1,081,959,313	14,511,170	4,613,258
LP6005442-B10	CLEAN	38.5	1,188,153,973	15,480,692	5,663,335
LP6005441-B11	CLEAN	31.4	978,701,100	10,893,612	5,888,699
LP6005442-B01	CLEAN	36.0	1,120,594,847	28,059,004	4,669,396
LP6005441-B02	CLEAN	35.0	1,087,255,527	7,538,860	5,645,256
SS6004478	CLEAN	41.1	1,278,416,598	14,477,190	4,684,206
LP6005442-A02	CLEAN	31.6	979,843,000	9,659,734	5,585,768
LP6005441-A03	CLEAN	36.1	1,120,280,917	8,779,936	4,665,082
LP6005443-A01	CLEAN	44.7	1,401,485,571	24,188,424	5,634,441
LP6005441-C01	CLEAN	39.5	1,229,513,422	9,803,092	4,664,121
LP6005592-D01	CLEAN	41.7	1,285,926,335	13,450,762	4,629,976
LP6005592-B03	CLEAN	42.4	1,316,856,631	20,445,902	4,749,335
LP6005519-B04	CLEAN	48.6	1,497,121,528	18,476,552	4,783,888
SS6004470	CLEAN	35.7	1,116,545,170	16,501,980	5,529,744

SDGP Sample	Contam Status	WGS Coverage	Mapped Reads	Unmapped Reads	Total Variants
LP6005442-H12	CLEAN	40.4	1,245,326,932	14,598,364	4,671,793
LP6005519-A05	CLEAN	42.8	1,318,753,391	24,479,388	4,779,911
LP6005592-A04	CLEAN	32.1	1,003,436,510	15,946,556	4,626,001
LP6005443-H11	CLEAN	43.4	1,347,677,569	15,745,408	4,706,734
LP6005519-C03	CLEAN	48.5	1,500,432,360	40,559,652	4,627,604
LP6005592-C02	CLEAN	44.7	1,387,691,266	15,960,464	4,684,153
LP6005441-D08	CLEAN	34.5	1,069,314,087	8,970,740	4,570,450
LP6005443-D06	CLEAN	40.9	1,269,014,748	12,933,080	4,650,786
LP6005442-H03	CLEAN	35.5	1,096,869,811	4,863,374	4,618,711
LP6005443-H02	CLEAN	36.1	1,112,028,522	11,767,174	4,633,449
LP6005441-F06	CLEAN	32.0	994,718,130	4,953,750	4,662,056
LP6005443-F04	CLEAN	36.2	1,115,551,983	19,809,952	4,648,845
LP6005443-B08	CLEAN	38.3	1,189,944,166	25,330,416	4,565,157
LP6005443-E05	CLEAN	41.8	1,289,301,913	22,639,232	4,724,291
LP6005441-E07	CLEAN	31.7	987,829,771	18,564,624	5,595,055
LP6005443-G03	CLEAN	43.3	1,332,428,817	15,244,518	4,549,099
LP6005441-G05	CLEAN	39.8	1,234,087,186	8,297,524	4,613,206
LP6005441-C09	CLEAN	37.3	1,155,938,178	6,565,332	4,647,209
LP6005442-G04	CLEAN	37.2	1,148,622,365	2,295,356	4,670,852
LP6005443-C07	CLEAN	38.8	1,215,810,180	23,279,386	4,506,462
LP6005443-E10	CLEAN	39.8	1,240,752,290	21,096,936	4,703,763
LP6005442-E11	CLEAN	33.4	1,039,309,439	2,433,330	5,554,173
LP6005442-F10	CLEAN	42.5	1,312,602,558	21,781,478	4,680,248
LP6005441-D04	CLEAN	36.1	1,124,448,938	7,985,136	4,640,191
LP6005443-D02	CLEAN	43.8	1,361,492,612	17,803,082	4,702,412
LP6005442-D03	CLEAN	39.9	1,234,258,145	94,947,964	4,703,677
LP6005441-F02	CLEAN	38.9	1,211,258,036	7,798,538	4,761,534
LP6005442-F01	CLEAN	37.1	1,162,319,875	34,036,706	4,637,206
LP6005443-B04	CLEAN	46.9	1,448,776,029	14,697,502	4,661,124
LP6005441-B06	CLEAN	74.4	2,314,771,939	38,612,508	4,746,752
LP6005443-E01	CLEAN	53.8	1,670,472,697	23,708,924	4,681,929
LP6005441-E03	CLEAN	38.9	1,213,596,274	4,542,798	4,717,045
LP6005442-C04	CLEAN	36.0	1,108,611,437	6,214,786	4,740,308
LP6005441-C05	CLEAN	39.0	1,222,024,097	5,556,456	4,640,112
LP6005443-C03	CLEAN	43.9	1,354,383,860	15,973,410	4,722,689
LP6005519-D06	CLEAN	41.6	1,296,059,514	88,857,724	4,543,454
LP6005677-D01	CLEAN	36.2	1,123,111,863	21,473,182	4,514,363
LP6005592-H01	CLEAN	48.0	1,482,258,748	16,763,148	4,788,028
LP6005519-F04	CLEAN	44.0	1,358,094,624	21,601,408	4,780,164
LP6005592-F03	CLEAN	46.3	1,431,895,657	17,517,456	4,738,274
LP6005519-E05	CLEAN	45.7	1,410,565,496	20,997,980	4,735,700
LP6005519-G03	CLEAN	47.7	1,476,193,580	23,485,190	4,821,678
LP6005441-H08	CLEAN	37.2	1,153,768,072	4,222,228	5,023,854
LP6005443-F08	CLEAN	39.7	1,233,375,506	23,394,480	4,597,606
LP6005442-F09	CLEAN	35.1	1,083,780,786	4,483,452	5,658,286
LP6005443-E09	CLEAN	38.2	1,185,796,979	19,983,736	4,704,117
LP6005443-G07	CLEAN	40.7	1,269,382,467	24,186,186	4,607,671
LP6005441-G09	CLEAN	38.9	1,203,231,939	3,866,860	4,755,841

Table S2. Top 20 highest ranking genes by continent, ordered by positive SHAP value.

African	American	Ashkenazi Jewish	East Asian	European	South Asian
ANKRD45, TEX50	KRTAP19-8	FGFBP2	POU2F3, RP11-778O17.4	ERP29	HSD3B7
IRF2BP2	FRMD8	TRDMT1	ERP29	ACAD10	CD320
GPR152	C2orf42	LCORL	EIF2AK2	KDSR	EML5
AX748340, PRKAG3	NCKIPSD	HBB, DL074624	ZNF140	LGALS16	ITIH4
PTK6	GRK2	CCL26	MEOX2, AC005550.3	HHAT	TAS2R3
TRPS1	GCC2, GCC2-AS1	PPARGC1A	SLC18A3	HECTD4	CPEB2
LPP, MIR28	KCNIP3	PNMA8A	HECTD4	IFT52	KDSR
GUCA1ANB	SSH3	UNC5B, UNC5B-AS1	EFCAB12	H1-8	IFNGR1
SLC28A2	ANXA4, AK125871	SLC39A12, SLC39A12-AS1	ZNF251	GTPBP10	MTFMT
FRG1	DISP2	DRD1	FUT2	PNMA8A	MFN1
ADAMTS6	TNFSF13B	MYRF, MYRF-AS1	FAM209B	CYHR1	SIGLEC11
CDC14B	TCIRG1	WDSUB1	AX747444, ZBTB43	OR5A2	MIR31HG, IFNE, MIR31
GRTP1	FARP2, AK055601, STK25	ANKMY2	PATZ1	MRPL9	GFOD2
PLEC, MIR661	SLC12A9	LANCL2	LIMK2	TARDBP	AJAP1
ARHGEF1	GSDMB	EXOSC5	ACAD10	SHISA2	RANBP6
PTPN18	BNIP5	KCNS3	SCARB1, JB074994	EYS, LOC441155	PNOC
STAT5B	NDUFAF7	FOXO3	THOC5	SCN7A	ITIH3
PACS2	BPIFB1	IQSEC3, RP11-598F7.4	MED13	SIRT2	VPS25
KRTAP4-4	MLKL	ZDHHC4, DQ579268	GLB1, TMPPE	NCKIPSD	EEA1
HMBS	ANO7	DNMBP, DNMBP-AS1	FAM118B, RNU4-86P, SRPRA	MFSD14B	IL20RA

Table S3. SNVStory ancestry inference validation. In-house clinical research dataset with self-reported race (W: White; B: Black or African; A: Asian; M: Multi-racial/Bi-racial; U: Unknown/Unspecified) and ethnicity (HL: Hispanic or Latino; Non-HL: Non-Hisplanic or Latino; Unk: Unknown/Unreported Ethnicity) and genetic ancestry prediction for gnomAD, the 1000 Genomes Project (1kGP), and the Simons Genome Diversity Project (SGDP) models (Afr: African/African American; Amr: Latino/Admixed American; Asj: Ashkenazi Jewish; Eas: East Asian, Eur: European/West Eurasia; Sas: South Asia; Cas: Central Asia/Siberia). Each row is an individual patient. Shading indicates model agreement (green), disagreement (red) or mixed race (orange).

Subject	Ethnicity	gnomAD	1kGP	SGDP
**Asian**				
A1	Non-HL	Eas	Eas	Eas
A1	Non-HL	Eas	Eas	Eas
A1	Non-HL	Eas	Eas	Eas
A1	Non-HL	Sas	Sas	Sas
A1	Non-HL	Sas	Sas	Sas
A1	Non-HL	Sas	Sas	Sas
**Black or African American**				
B1	Non-HL	Afr	Afr	Afr
B2	Non-HL	Afr	Afr	Afr
B3	Non-HL	Afr	Afr	Afr
B4	Non-HL	Afr	Afr	Afr
B5	Non-HL	Afr	Afr	Afr
B6	Non-HL	Afr	Afr	Afr
B7	Non-HL	Afr	Afr	Afr
B8	Non-HL	Afr	Afr	Afr
B9	Non-HL	Afr	Afr	Afr
B10	Non-HL	Afr	Afr	Afr
B11	Non-HL	Afr	Afr	Afr
B12	Non-HL	Afr	Afr	Afr
B13	Non-HL	Afr	Afr	Afr
B14	Non-HL	Afr	Afr	Afr
B15	Non-HL	Afr	Afr	Afr
B16	Non-HL	Afr	Afr	Afr
B17	Non-HL	Afr	Afr	Afr
B18	Non-HL	Afr	Afr	Afr
B19	Non-HL	Afr	Afr	Afr
B20	Non-HL	Afr	Amr	Afr
B21	HL	Amr	Amr	Afr
B22	HL	Amr	Amr	Afr
**White**				
W1	Unk	Eur	Eur	Eur
W2	Unk	Eur	Eur	Eur
W3	Unk	Eur	Eur	Eur
W4	Non-HL	Afr	Amr	Afr
W5	Non-HL	Amr	Amr	Eur
W6	Non-HL	Asj	Eur	Eur
W7	Non-HL	Eas	Amr	Sas
W8	Non-HL	Eas	Amr	Sas
W9	Non-HL	Eur	Amr	Eur
W10	Non-HL	Eur	Amr	Eur
W11	Non-HL	Eur	Amr	Eur
W12	Non-HL	Eur	Amr	Eur
W13	Non-HL	Eur	Eur	Eur
W14	Non-HL	Eur	Eur	Eur
W15	Non-HL	Eur	Eur	Eur
W16	Non-HL	Eur	Eur	Eur
W17	Non-HL	Eur	Eur	Eur
W18	Non-HL	Eur	Eur	Eur
W19	Non-HL	Eur	Eur	Eur
W20	Non-HL	Eur	Eur	Eur
W21	Non-HL	Eur	Eur	Eur
W22	Non-HL	Eur	Eur	Eur
W23	Non-HL	Eur	Eur	Eur
W24	Non-HL	Eur	Eur	Eur
W25	Non-HL	Eur	Eur	Eur
W26	Non-HL	Eur	Eur	Eur
W27	Non-HL	Eur	Eur	Eur
W28	Non-HL	Eur	Eur	Eur
W29	Non-HL	Eur	Eur	Eur
W30	Non-HL	Eur	Eur	Eur
W31	Non-HL	Eur	Eur	Eur
W32	Non-HL	Eur	Eur	Eur
W33	Non-HL	Eur	Eur	Eur
W34	Non-HL	Eur	Eur	Eur
W35	Non-HL	Eur	Eur	Eur
W36	Non-HL	Eur	Eur	Eur
W37	Non-HL	Eur	Eur	Eur
W38	Non-HL	Eur	Eur	Eur
W39	Non-HL	Eur	Eur	Eur
W40	Non-HL	Eur	Eur	Eur
W41	Non-HL	Eur	Eur	Eur
W42	Non-HL	Eur	Eur	Eur
W43	Non-HL	Eur	Eur	Eur
W44	Non-HL	Eur	Eur	Eur
W45	Non-HL	Eur	Eur	Eur
W46	Non-HL	Eur	Eur	Eur
W47	Non-HL	Eur	Eur	Eur
W48	Non-HL	Eur	Eur	Eur
W49	Non-HL	Eur	Eur	Eur
W50	Non-HL	Eur	Eur	Eur
W51	Non-HL	Eur	Eur	Eur
W52	Non-HL	Eur	Eur	Eur
W53	Non-HL	Eur	Eur	Eur
W54	Non-HL	Eur	Eur	Eur
W55	Non-HL	Eur	Eur	Eur
W56	Non-HL	Eur	Eur	Eur
W57	Non-HL	Eur	Eur	Eur
W58	Non-HL	Eur	Eur	Eur
W59	Non-HL	Eur	Eur	Eur
W60	Non-HL	Eur	Eur	Eur
W61	Non-HL	Eur	Eur	Eur
W62	Non-HL	Eur	Eur	Eur
W63	Non-HL	Eur	Eur	Eur
W64	Non-HL	Eur	Eur	Eur
W65	Non-HL	Eur	Eur	Eur
W66	Non-HL	Eur	Eur	Eur
W67	Non-HL	Eur	Eur	Eur
W68	Non-HL	Eur	Eur	Eur
W69	Non-HL	Eur	Eur	Eur
W70	Non-HL	Eur	Eur	Eur
W71	Non-HL	Eur	Eur	Eur
W72	Non-HL	Eur	Eur	Eur
W73	Non-HL	Eur	Eur	Eur
W74	Non-HL	Eur	Eur	Eur
W75	Non-HL	Eur	Eur	Eur
W76	Non-HL	Eur	Eur	Eur
W77	Non-HL	Eur	Eur	Eur
W78	Non-HL	Eur	Eur	Eur
W79	Non-HL	Eur	Eur	Eur
W80	Non-HL	Eur	Eur	Eur
W81	Non-HL	Eur	Eur	Eur
W82	Non-HL	Eur	Eur	Eur
W83	Non-HL	Eur	Eur	Eur
W84	Non-HL	Eur	Eur	Eur
W85	Non-HL	Eur	Eur	Eur
W86	Non-HL	Eur	Eur	Eur
W87	Non-HL	Eur	Eur	Eur
W88	Non-HL	Eur	Eur	Eur
W89	Non-HL	Eur	Eur	Eur
W90	Non-HL	Eur	Eur	Eur
W91	Non-HL	Eur	Eur	Eur
W92	Non-HL	Eur	Eur	Eur
W93	Non-HL	Eur	Eur	Eur
W94	Non-HL	Eur	Eur	Eur
W95	Non-HL	Eur	Eur	Eur
W96	Non-HL	Eur	Eur	Eur
W97	Non-HL	Eur	Eur	Eur
W98	Non-HL	Eur	Eur	Eur
W99	Non-HL	Eur	Eur	Eur
W100	Non-HL	Eur	Eur	Eur
W101	Non-HL	Eur	Eur	Eur
W102	Non-HL	Eur	Eur	Eur
W103	Non-HL	Eur	Eur	Eur
W104	Non-HL	Eur	Eur	Eur
W105	Non-HL	Eur	Eur	Eur
W106	Non-HL	Eur	Eur	Eur
W107	Non-HL	Eur	Eur	Eur
W108	Non-HL	Eur	Eur	Eur
W109	Non-HL	Eur	Eur	Eur
W110	Non-HL	Eur	Eur	Eur
W111	Non-HL	Eur	Eur	Eur
W112	Non-HL	Eur	Eur	Eur
W113	Non-HL	Eur	Eur	Eur
W114	Non-HL	Eur	Eur	Eur
W115	Non-HL	Eur	Eur	Eur

Subject	Ethnicity	gnomAD	1kGP	SGDP
W116	Non-HL	Eur	Eur	Eur
W117	Non-HL	Eur	Eur	Eur
W118	Non-HL	Eur	Eur	Eur
W119	Non-HL	Eur	Eur	Eur
W120	Non-HL	Eur	Eur	Eur
W121	Non-HL	Eur	Eur	Eur
W122	Non-HL	Eur	Eur	Eur
W123	Non-HL	Eur	Eur	Eur
W124	Non-HL	Eur	Eur	Eur
W125	Non-HL	Eur	Eur	Eur
W126	Non-HL	Eur	Eur	Eur
W127	Non-HL	Eur	Eur	Eur
W128	Non-HL	Eur	Eur	Eur
W129	Non-HL	Eur	Eur	Eur
W130	Non-HL	Eur	Eur	Eur
W131	Non-HL	Eur	Eur	Eur
W132	Non-HL	Eur	Eur	Eur
W133	Non-HL	Eur	Eur	Eur
W134	Non-HL	Eur	Eur	Eur
W135	Non-HL	Eur	Eur	Eur
W136	Non-HL	Eur	Eur	Eur
W137	Non-HL	Eur	Eur	Eur
W138	Non-HL	Eur	Eur	Eur
W139	Non-HL	Eur	Eur	Eur
W140	Non-HL	Eur	Eur	Eur
W141	Non-HL	Eur	Eur	Eur
W142	Non-HL	Eur	Eur	Eur
W143	Non-HL	Eur	Eur	Eur
W144	Non-HL	Eur	Eur	Eur
W145	Non-HL	Eur	Eur	Eur
W146	Non-HL	Eur	Eur	Eur
W147	Non-HL	Eur	Eur	Eur
W148	Non-HL	Eur	Eur	Eur
W149	Non-HL	Eur	Eur	Eur
W150	Non-HL	Eur	Eur	Eur
W151	Non-HL	Eur	Eur	Eur
W152	Non-HL	Eur	Eur	Eur
W153	Non-HL	Eur	Eur	Eur
W154	Non-HL	Eur	Eur	Eur
W155	Non-HL	Eur	Eur	Eur
W156	Non-HL	Eur	Eur	Eur
W157	Non-HL	Eur	Eur	Eur
W158	Non-HL	Eur	Eur	Eur
W159	Non-HL	Eur	Eur	Eur
W160	Non-HL	Eur	Eur	Eur
W161	Non-HL	Eur	Eur	Eur
W162	Non-HL	Eur	Eur	Eur
W163	Non-HL	Eur	Eur	Eur
W164	Non-HL	Eur	Eur	Eur
W165	Non-HL	Eur	Eur	Eur
W166	Non-HL	Eur	Eur	Eur
W167	Non-HL	Eur	Eur	Eur
W168	Non-HL	Eur	Eur	Eur
W169	Non-HL	Eur	Eur	Eur
W170	Non-HL	Eur	Eur	Eur
W171	Non-HL	Eur	Eur	Eur
W172	Non-HL	Eur	Eur	Eur
W173	Non-HL	Eur	Eur	Eur
W174	Non-HL	Eur	Eur	Eur
W175	Non-HL	Eur	Eur	Eur
W176	Non-HL	Eur	Eur	Eur
W177	Non-HL	Eur	Eur	Eur
W178	Non-HL	Eur	Eur	Eur
W179	Non-HL	Eur	Eur	Eur
W180	Non-HL	Eur	Eur	Eur
W181	Non-HL	Eur	Eur	Eur
W182	Non-HL	Eur	Eur	Eur

Subject	Ethnicity	gnomAD	1kGP	SGDP
W183	Non-HL	Eur	Eur	Eur
W184	Non-HL	Eur	Eur	Eur
W185	Non-HL	Eur	Eur	Eur
W186	Non-HL	Eur	Eur	Eur
W187	Non-HL	Eur	Eur	Eur
W188	Non-HL	Eur	Eur	Eur
W189	Non-HL	Eur	Eur	Eur
W190	Non-HL	Eur	Eur	Eur
W191	Non-HL	Eur	Eur	Eur
W192	Non-HL	Eur	Eur	Eur
W193	Non-HL	Eur	Eur	Eur
W194	Non-HL	Eur	Eur	Eur
W195	Non-HL	Eur	Eur	Eur
W196	Non-HL	Eur	Eur	Eur
W197	Non-HL	Eur	Eur	Eur
W198	Non-HL	Eur	Eur	Eur
W199	Non-HL	Eur	Eur	Eur
W200	Non-HL	Eur	Eur	Eur
W201	Non-HL	Eur	Eur	Eur
W202	Non-HL	Eur	Eur	Eur
W203	Non-HL	Eur	Eur	Eur
W204	Non-HL	Eur	Eur	Eur
W205	Non-HL	Eur	Eur	Eur
W206	Non-HL	Eur	Eur	Eur
W207	Non-HL	Eur	Eur	Eur
W208	Non-HL	Eur	Eur	Eur
W209	Non-HL	Eur	Eur	Eur
W210	Non-HL	Eur	Eur	Eur
W211	Non-HL	Eur	Eur	Eur
W212	Non-HL	Eur	Eur	Eur
W213	Non-HL	Eur	Eur	Eur
W214	Non-HL	Eur	Eur	Eur
W215	Non-HL	Eur	Eur	Eur
W216	Non-HL	Eur	Eur	Eur
W217	Non-HL	Eur	Eur	Eur
W218	Non-HL	Eur	Eur	Eur
W219	Non-HL	Sas	Sas	Sas
W220	HL	Afr	Amr	Eur
W221	HL	Amr	Amr	Cas
W222	HL	Amr	Amr	Eur
W223	HL	Amr	Amr	Eur
W224	HL	Amr	Amr	Eur
W225	HL	Amr	Amr	Sas
W226	HL	Amr	Amr	Sas
W227	HL	Amr	Amr	Sas
W228	HL	Amr	Amr	Sas
W229	HL	Eur	Amr	Eur
W230	HL	Eur	Amr	Eur
W231	HL	Eur	Amr	Eur
**Multi-racial/Bi-racial**				
M1	Unk	Afr	Afr	Afr
M2	Unk	Afr	Amr	Afr
M3	Unk	Afr	Amr	Afr
M4	Unk	Eur	Eur	Eur
M5	Non-HL	Afr	Afr	Afr
M6	Non-HL	Afr	Afr	Afr
M7	Non-HL	Afr	Amr	Afr
M8	Non-HL	Afr	Amr	Afr
M9	Non-HL	Afr	Amr	Afr
M10	Non-HL	Afr	Amr	Afr
M11	Non-HL	Afr	Amr	Afr
M12	Non-HL	Afr	Amr	Afr
M13	Non-HL	Afr	Amr	Afr
M14	Non-HL	Afr	Amr	Eur
M15	Non-HL	Eur	Amr	Eur
M16	Non-HL	Eur	Amr	Eur
M17	Non-HL	Eur	Eur	Eur

Subject	Ethnicity	gnomAD	1kGP	SGDP
M18	Non-HL	Eur	Eur	Eur
M19	Non-HL	Eur	Eur	Eur
M20	HL	Afr	Afr	Afr
M21	HL	Afr	Amr	Afr
M22	HL	Amr	Amr	Eur
M23	HL	Eas	Amr	Eur
M24	HL	Eur	Amr	Afr
M25	HL	Eur	Amr	Eur
M26	HL	Eur	Amr	Eur
M27	HL	Eur	Amr	Eur
M28	HL	Eur	Amr	Eur
**Unknown/Unreported Race**				
U1	HL	Amr	Amr	Cas
U2	HL	Amr	Amr	Cas
U3	HL	Amr	Amr	Cas
U4	HL	Amr	Amr	Eur
U5	HL	Amr	Amr	Eur
U6	HL	Eur	Amr	Eur
